# Guilt and Proneness to Shame: Unethical Behaviour in Vulnerable and Grandiose Narcissism

**DOI:** 10.5964/ejop.v14i1.1355

**Published:** 2018-03-12

**Authors:** Pauline Georgees Poless, Linda Torstveit, Ricardo Gregorio Lugo, Marita Andreassen, Stefan Sütterlin

**Affiliations:** a Department of Psychosocial Science, University of Bergen, Bergen, Norway; b Department of Psychology, Inland Norway University of Applied Sciences, Lillehammer, Norway; c Department of Psychology, University of Copenhagen, Copenhagen, Denmark; d CHTD Research Group, Division of Clinical Neuroscience, Oslo University Hospital, Oslo, Norway; e Faculty of Health and Welfare Sciences, Østfold University College, Fredrikstad, Norway; Department of Psychology, Webster University Geneva, Geneva, Switzerland; Edinburgh Napier University, Edinburgh, United Kingdom

**Keywords:** shame and guilt proneness in narcissism, vulnerable narcissism, grandiose narcissism, unethical decision making in narcissism, unethical behaviour

## Abstract

Narcissists are described as individuals with dysfunctional personality traits such as lack of psychological awareness and empathy. Theories of ethical behaviour assume that unethical actions trigger moral emotions of guilt and shame. Currently, there is a lack of knowledge on moral emotions as dispositional traits and their potential influences on behaviour in individuals with narcissistic traits. The present study examined vulnerable and grandiose narcissism’s differences in the propensity to experience guilt and shame as a proneness, across a range of personal transgressions. Guilt proneness was measured by negative evaluation of unethical behaviour, and whether this evaluation could influence reparation of tendencies of unethical action in vulnerable and grandiose narcissism. Shame proneness was investigated by negative evaluation of the self, and then whether the previous tendency could affect unethical decision making and behaviour (e.g., hiding), in vulnerable and grandiose narcissism. Two hundred and sixteen participants responded to the Guilt and Shame Proneness Scale, the Narcissistic Personality Inventory Scale and the Hypersensitive Narcissism Scale in an online questionnaire. Findings indicate that grandiose narcissism was negatively associated with guilt proneness, and the relation between the vulnerable narcissism and guilt proneness was negative. Additionally, the results confirm a negative association between grandiose narcissism and shame proneness, especially related to the subscale ‘shame negative self-evaluation’. Furthermore, guilt and shame proneness explained 20% of the variance in vulnerable narcissism and 11% in grandiose narcissism. This research indicates that both vulnerable and grandiose narcissism have the tendency to make unethical decisions, and they are more likely to enact in unethical behaviour. These findings are relevant for the detection of narcissistic individual’s propensity to act unethically in social context.

## Narcissism: The Grandiose and Vulnerable Type

The term narcissism describes personality traits of individuals with a self-centric orientation, followed by thoughts of unlimited power and success, and an excessive need of encouragement and special treatment (American Psychiatric Association, 2013). Narcissistic personality disorder has numerous detrimental implications for social adaptation and interaction (Beck et al., 2001; Dickinson & Pincus, 2003; Schoenleber, Sadeh, & Verona, 2011). While individuals with strong narcissistic traits often show assertive and arrogant characteristics, they often experience anxiety, suspiciousness and hypersensitivity (Murray, 1938). Narcissistic qualities, such as a pronounced sense of entitlement and lack of empathy, coexist in line with increased vulnerability, the feeling of inferiority, discontentment with life and a higher risk of depression (Kernberg, 1975). More recent research on these seemingly contradictory characteristics of narcissistic personalities led to the identification of the subtypes vulnerable and grandiose narcissism with different expressions of cognitive and behavioural features (Cain, Pincus, & Ansell, 2008; Pincus & Lukowitsky, 2010). The grandiose subtype is characterized by both internal and external self-defensive attributes about oneself, a need for attention and special treatment, a high esteem of self-meaning, extraversion and social dominance, and is interpersonally manipulative (e.g., exploits others to gain personal goals). In contrast, the vulnerable subtype often suffers from inferiority, negative emotionality and dependency on external sources of admiration, high level of vigilance; but can also be characterized by entitlement and grandiose fantasy (Ackerman, Hands, Donnellan, Hopwood, & Witt, 2017; Cain et al., 2008, Pincus & Lukowitsky 2010; Ronningstam, 2011). Consequently, grandiose narcissism is associated with a dominating and exploitative social style, as opposed to vulnerable narcissism that displays higher proneness to feelings of inadequacy in social contexts, hypersensitivity based on others evaluation, and high aggression (Dickinson & Pincus, 2003; Schoenleber et al., 2011).

## Narcissism and Moral Emotions

The psychological literature often connects narcissistic personality traits to maladaptive behaviour and social dysfunction, e.g., aggression, low agreeableness, impulsivity, psychopathy and lack of empathy (Hepper, Hart, Meek, Cisek, & Sedikides, 2014; Holtzman, Vazire, & Mehl, 2010; Schoenleber et al., 2011; Vazire & Funder, 2006). Lack of moral emotions such as guilt and shame might provide a possible reason for the observed maladaptive behaviour. A growing body of research supports the notion that individuals who are more inclined to ethical behaviours often show a lower threshold for experiencing guilt (Cohen, Panter, & Turan, 2012; Katchadourian, 2010; Torstveit, Sütterlin, & Lugo, 2016). Moral emotions play a crucial role in deterring unethical and antisocial behaviour (Olthof, 2012; Xu, Bègue, & Bushman, 2012). Individuals who act in a manner inconsistent with their own moral ideals and standards, may experience negative emotions such as anger, aggression and/ or perception of guilt (Stuewig, Tangney, Heigel, Harty, & McCloskey, 2010). Studies suggest that individuals who act in accordance with moral emotions are less likely to commit criminal offenses and are more reliable to others (Stuewig & McCloskey, 2005).

Guilt and shame are characterized by feelings of distress arising in response to personal transgressions (Tangney & Dearing, 2002; Tangney, Stuewig, & Mashek, 2007; Wolf, Cohen, Panter, & Insko, 2010). Nevertheless, there are two differences between these emotional tendencies, the self-behaviour distinction and the public-private distinction (Cohen, Wolf, Panter, & Insko, 2011). Guilt arises from behaviours which lead to negative feelings about specific actions that the person has committed (Tracy & Robins, 2004) and is an emotional response that is directed against the act, (e.g., “I have done something wrong”). In contrast, shame is a negative emotion that originates from a person's self-evaluation (e.g., “I am a bad person”), which leads to an experience of negative emotions about the public self (Tracy & Robins, 2004). People who tend to experience shame are more likely to suffer from low self-esteem, high neuroticism, and personal distress. According to Tangney and Dearing (2002), individuals that score high in guilt are more likely to attempt to change their behaviour, while individuals low in guilt are more prone to retreat and conceal their negative actions. Narcissistic individuals, in particular the grandiose subtype, are negatively associated with guilt and shame (Czarna, 2014; Wright, O’Leary, & Balkin, 1989). Furthermore, the vulnerable dimension of narcissism is positively associated with shame (Freis, Brown, Carroll, & Arkin, 2015; Malkin, Barry, & Zeigler-Hill, 2011), while there is a lack of knowledge regarding to the relationship between the vulnerable narcissism and guilt.

Both shame and guilt have been described as dispositions or proneness that may influence individuals’ ethical behaviour (Cohen et al., 2011). The concept of the proneness to experience guilt is usually operationalized by the assessment of an individual’s evaluation of previously committed negative behaviour and the tendency to repair it. Shame proneness is operationalized as negative self-evaluation and subsequent withdrawal when a transgression is public and the tendency to conceal it (Cohen et al., 2011).

While previous research primarily investigated the relation between narcissistic individuals and moral emotions typically operationalized as a subjectively experienced momentary state (e.g. Giammarco & Vernon, 2015; Thomaes, Bushman, Stegge, & Olthof, 2008; Wright et al., 1989), there is a lack of research on the effect of moral emotions and prediction on ethical behaviour in these individuals. It remains unclear how associations between moral emotions and ethical behaviour affect the different subtypes of narcissisms. This study attempts to address some of the difficulties in current research, and aims to explore possible associations between proneness to experience moral emotions, based on guilt and shame, and ethical behaviour in individuals with narcissistic traits (e.g., vulnerable and grandiose narcissism).

## Hypotheses

Because vulnerable and grandiose narcissism usually report different associations to shame (Freis et al., 2015; Malkin et al., 2011; Wright et al., 1989), as well as the exploration of the relationship to guilt is still unexplored, it seems reasonable to investigate both subtypes separately. Since vulnerable narcissism is usually associated with low levels of self-esteem, (a) we expect a positive association between the vulnerable narcissism subtype and shame based on negative self-evaluation. Since narcissistic individuals tend to report a reduced ability to feel guilt and usually report low on empathy (Hepper, Hart, Meek, et al., 2014; Wright et al., 1989), (b) we further expect a negative association between vulnerable narcissism and guilt negative behaviour evaluation, as well as a negative association between the vulnerable narcissism and guilt repair. We further expect (c) a negative relation between grandiose narcissism and guilt negative behaviour evaluation, and a negative association between the grandiose narcissism and guilt repair. Due to the nature of narcissistic behaviour, we (d) expect that guilt negative behaviour evaluation and shame negative self-evaluation will mediate ethical or unethical behaviour of narcissism.

## Method

### Research Design and Procedure

Data were acquired via online questionnaires. Participation was anonymous and voluntary; no compensation was given. Questionnaires were distributed and completed by using the online survey system *SoSciSurvey* (Leiner, 2014, www.soscisurvey.de). The survey could be answered in two languages (Norwegian and English). The study has been approved by the Norwegian Social Science Data Services (NSD; project nr. 45524).

Participants (*N* = 216; 74.5% female) were recruited through social media and from universities. 40.7% reported Norwegian as their first language and 59.3% other languages such as English, German, Arabic, Russian, Spanish, Danish, etc. Mean age was 26.70 (*SD* = 11.3, range 15 - 70 years). Eleven participants did not deliver complete questionnaires, presumably due to the length of the survey (15 minutes). This is the reason why this article will report two different sample sizes later in this section.

### Questionnaires

#### Narcissistic Personality Inventory

Levels of grandiose narcissism were measured with the Narcissistic Personality Inventory (NPI; Ames, Rose, & Anderson, 2006). The scale is based on DSM IV criteria for narcissistic personality disorder, but was developed to assess individual degrees of non-clinical narcissism in the general public. This study applied shorter version of the NPI measures the most characteristic components of grandiose narcissism, which are authority, superiority, exhibitionism, selfishness and feeling of justice (Ames et al., 2006). Each of the 16 items consists of two statements. Exemplary items are “I like to be in the center of attention” and “I feel uncomfortable when I’m in center of attention”. Reliability was reported to be Cronbach’s alpha = .86 (Ames et al., 2006).

#### Hypersensitive Narcissism Scale

The Hypersensitive Narcissism Scale (HSNS, Hendin & Cheek, 1997) was used to assess vulnerable narcissism. The HSNS is based on the Narcissism Scale by Murray (1938) and consists of 10 items responded to on a 5- point Likert scale. An exemplary item is “I dislike being with a group unless I know that I am appreciated by at least one of those present.”, where the participant is asked to rate from 1 (very uncharacteristic/untrue/strongly disagree) to 5 (very characteristics/true/strongly agree). The study of Hendin and Cheek (1997) reported a Cronbach’s alpha of .76.

#### Guilt and Shame Proneness Scale

Guilt and shame proneness was measured by Guilt and Shame Proneness Scale (GASP; Cohen et al., 2011). GASP consists of 4 different subscales: Guilt negative behaviour evaluation, guilt repair, shame negative self-evaluation and shame withdraw. It has 16 items intended to ask the respondents to imagine themselves in different situations, and afterwards ask what is the likelihood they would act, feel or think in a certain manner. An example of an item is “After realizing you have received too much change at a store, you decide to keep it because the salesclerk doesn't notice. What is the likelihood that you would feel uncomfortable about keeping the money?” The scale is a 7-point Likert scale, ranging from (1) very unlikely to (7) very likely. This scale is reported to have a Cronbach’s alpha of .60 (Cohen et al., 2011).

### Statistical Analysis

All statistical analysis was done by means of SPSS (Version 20, IBM Corporation, 2011). Assumption of normality and homogeneity of variance was tested prior to further analysis. A Pearson’s correlation was calculated for all relevant variables. A linear multiple regression was used to determine how much of the variance in the dependent variables vulnerable and grandiose narcissism could be explained by the independent variables guilt and shame proneness. Finally, mediation analyses were employed to understand if the relation between narcissism and ethical behaviour (guilt repair)/ and unethical behaviour (shame withdraw) may be affected by a mediator variable (e.g., guilt, negative behaviour evaluation, and shame, negative self-evaluation). The PROCESS model (Hayes, 2013) for SPSS is used to conduct the mediation analyses.

## Results

### Reliability Analysis

All psychometric instruments used in this study were tested for internal consistency. Shame withdraw showed low Cronbach’s alpha (.506), therefore this scale underwent an exploratory factor analysis. The factor analysis confirmed the one-factor structure with one factor exceeding an eigenvalue > 1 (1.615) explaining 40.38% of total variance. Cronbach’s alpha, descriptive statistics and reliability indices for the measurements are presented in Table 1.

**Table 1:** Descriptive Statistics (N = 216)

Variables	Cronbach’s α	Mean	*SD*	Min.	Max.
Grandiose narcissism	.711	3.62	2.84	0	14
Vulnerable narcissism	.663	28.73	5.10	15	47
Guilt-NBE	.759	21.98	5.10	4	28
Guilt-repair	.660	22.45	3.90	11	28
Shame-NSE	.713	22.99	4.30	7	28
Shame-withdraw	.506	11.79	4.02	4	24

*Note.* NBE = negative behaviour evaluation, NSE = negative self-evaluation.

### Correlation Between Variables

Pearson product-moment correlation was calculated to examine the relationships between vulnerable narcissism and shame proneness (negative self-evaluation), vulnerable narcissism and guilt proneness (negative self-evaluation and repair), and grandiose narcissism and guilt proneness (negative self-evaluation and repair). The results of this analytical procedure are shown in Table 2.

**Table 2:** Correlation Between Variables

Variable	Grandiose narcissism	Vulnerable narcissism
Guilt, negative behaviour evaluation	-.282**	-.175*
Guilt-repair	-.235**	-.227**
Shame, negative self-evaluation	-.305**	-.010
Shame-withdraw	-.068	.357**

*p < .01. **p < .001.

### Standard Multiple Regression and the Variance in the Grandiose Narcissism and Vulnerable Narcissism

To determine whether guilt proneness (negative self-evaluation and repair) would uniquely contribute to variance in grandiose narcissism, a standard multiple regression was performed, *F*(4, 197) = 6.093, *p* < .001, *R^2^* = .11 (11%). Guilt proneness has no unique contribution to explaining the variance in the grandiose narcissism (see Table 3).

**Table 3:** Standard Multiple Regression: Grandiose Narcissism

Predictor Variable	*B*	*SE B*	β
Guilt, negative behaviour evaluation	-.075	.054	-.134
Guilt-repair	-.031	.067	-.045
Shame, negative self-evaluation	-.125	.062	-.190
Shame-withdraw	-.047	.048	-.067

*Note. R^2^* = .110.

When it comes to vulnerable narcissism and guilt (negative self-evaluation and repair) - and shame proneness (negative self-evaluation), *F*(4, 203) = 12.435, *p* < .001, *R^2^* = .20 (20%), the results of standard multiple regression indicated that guilt proneness (repair) (3%, *R_Raj2_* = .184), had the only unique contribution to explaining the variance in the vulnerable narcissism (see Table 4).

**Table 4:** Standard Multiple Regression: Vulnerable Narcissism

Predictor Variable	*B*	*SE B*	β
Guilt, negative behaviour evaluation	-.139	.091	-.138
Guilt-repair	-.329	.112	-.249**
Shame, negative self-evaluation	.250	.104	.211
Shame-withdraw	.420	.080	.331**

*Note. R^2^* = .197.

*p < .01. **p < .001.

### Mediation Analysis

#### Grandiose Narcissism and Ethical Behaviour by Guilt Repair

**Figure 1:**
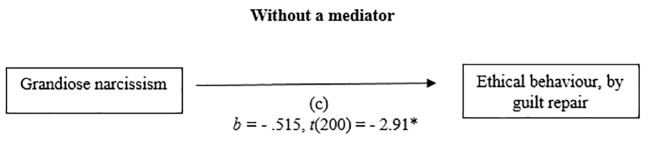
Mediation Analysis: Without a mediator.

**Figure 2:**
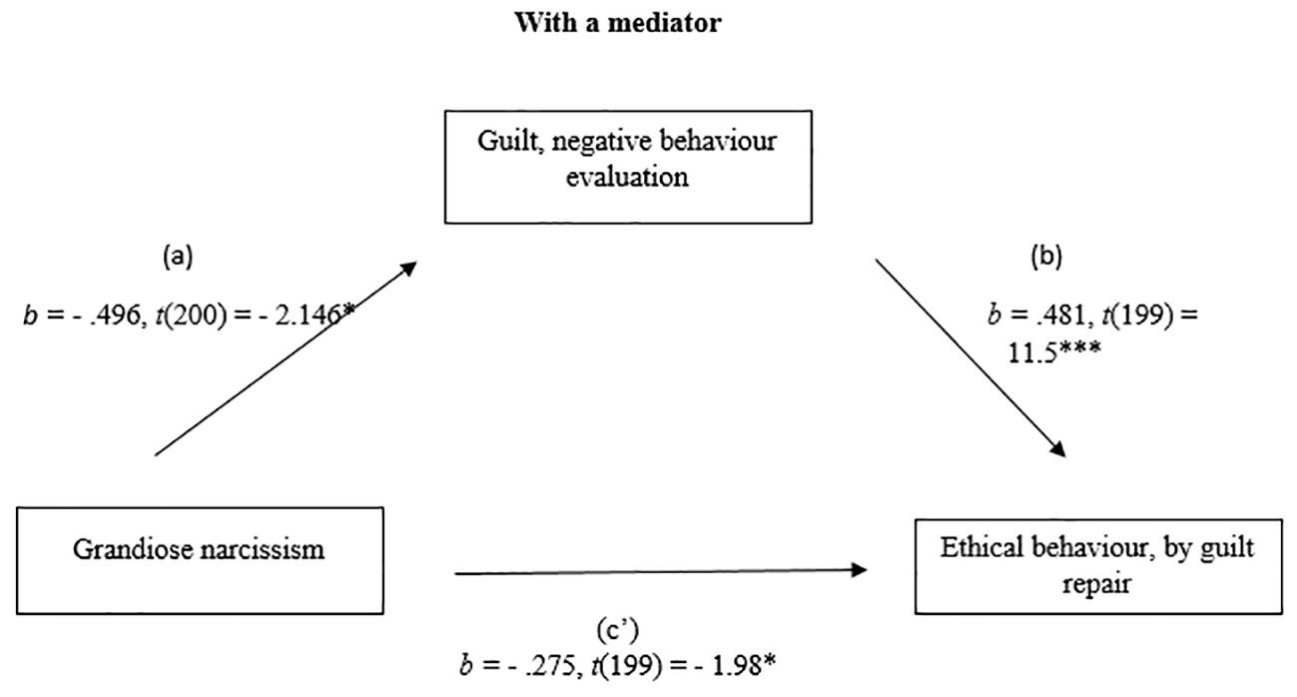
Mediation Analysis: With a mediator. *p < .05. **p < .01. ***p < .001.

The relationship between grandiose narcissism and ethical behaviour by guilt repair, was mediated by guilt, negative behaviour evaluation. The regression of the grandiose narcissism and ethical behaviour was significant, *b* = - .515, *t*(200) = - 2.91, *p* < .05, the regression of the grandiose narcissism predicting guilt, negative behaviour evaluation was also significant, *b* = - .496, *t*(200) = - 2.146, *p* = .03. The regression of the grandiose narcissism and guilt negative behaviour evaluation which combined predict unethical behaviour, were significant, *F*(2, 199) = 72.92, *p* < .001, *R^2^* = .42 (42%), *b* = .482, *t*(199) = 11.5, *p* < .001. A Sobel test was conducted and found mediation in the model *z* = - 2.10, *p* < .05, *ҝ^2^* = - .239. (See Figure 1 and 2).

#### Vulnerable Narcissism and Ethical Behaviour by Guilt Repair

**Figure 3:**
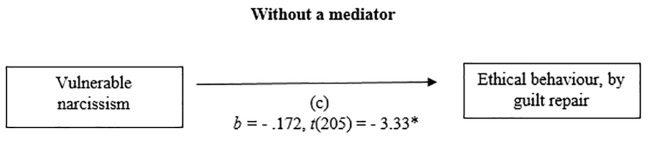
Mediation Analysis: Without a mediator.

**Figure 4:**
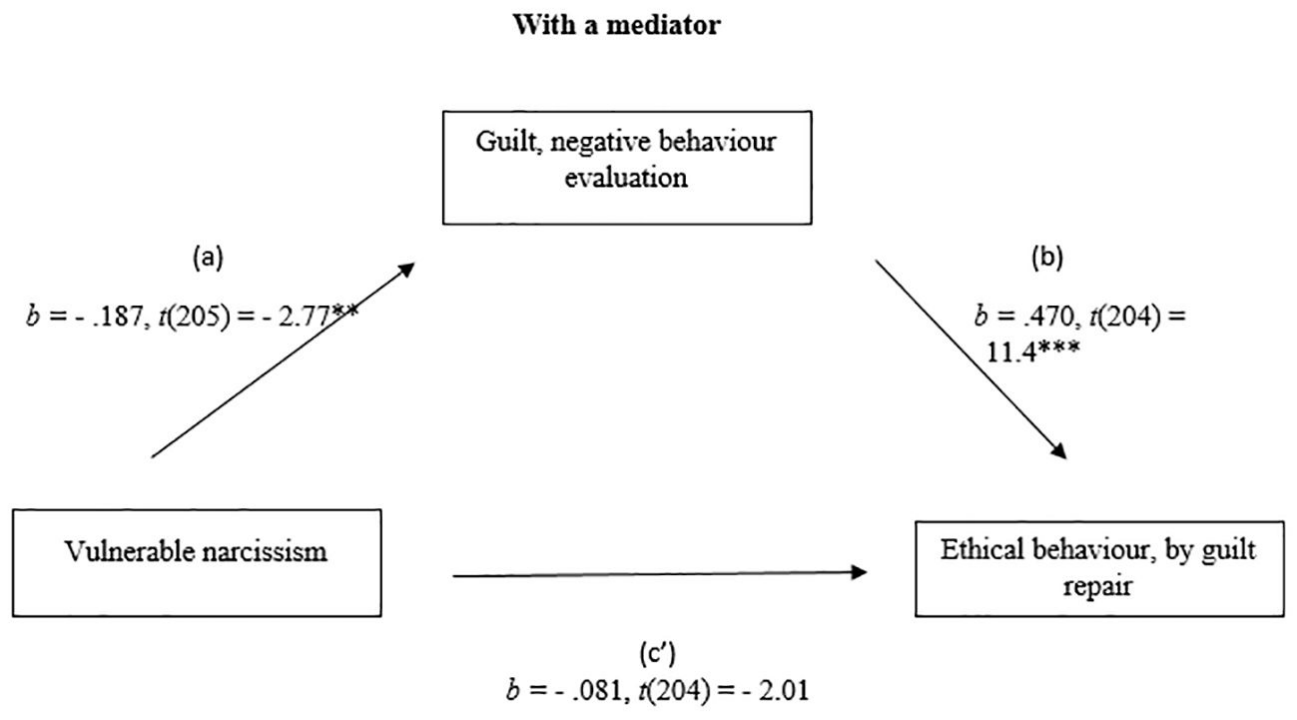
Mediation Analysis: With a mediator. *p < .05. **p < .01. ***p < .001.

The relationship between vulnerable narcissism and ethical behaviour by guilt repair was mediated by guilt, negative behaviour evaluation. The regression of the vulnerable narcissism and ethical behaviour was significant, *b* = - .172, *t*(205) = - 3.33, *p* < .01, the regression of the vulnerable narcissism predicting guilt, negative behaviour evaluation was also significant *b* = - .187, *t*(205) = - 2.77, *p* < .01. The regression of the vulnerable narcissism and guilt negative behaviour evaluation which together predict ethical behaviour, was significant, *F*(2.204) = 73.63, *p* < .001, *R^2^* = .42 (42%), *b* = .470, *t*(204) = 11.4, *p* < .001. A Sobel test was conducted and found mediation in the model *z* = - 2.68, *p* < .01, *ҝ^2^* = -.089 (see Figure 3 and 4).

#### Vulnerable Narcissism and Unethical Behaviour by Shame Withdraw

The relationship between vulnerable narcissism and ethical behaviour by shame withdraw was mediated by shame, negative self-evaluation. The regression of the vulnerable narcissism and ethical behaviour was significant, *b* = .287, *t*(205) = 5.488, *p* < .001, the regression of the vulnerable narcissism predicting shame, negative self-evaluation was not significant. The regression of the vulnerable narcissism and shame negative self-evaluation which together predict ethical behaviour, was significant, *F*(2, 204) = 15.213, *p* < .001, *R^2^* = .13 (13%), but not significant between shame, negative self-evaluation (mediator) and shame withdraw (unethical behaviour). A Sobel test was conducted, and the results were not significant, *z* = .106, *p* < .92, *ҝ^2^* = .0005.

#### Grandiose Narcissism and Unethical Behaviour by Shame Withdraw

Shame withdraw did not mediate the relationship between grandiose narcissism and ethical behaviour. A Sobel test was conducted and the result was as following, *z* = .303, *p* = .76, *ҝ^2^* = .0055.

## Discussion

### Guilt Proneness in Narcissistic Individuals

This study confirms that grandiose narcissism is negatively associated with guilt proneness (negative behaviour evaluation and repair). In addition, the vulnerable narcissism is also negatively associated with guilt proneness (negative behaviour evaluation and repair). If we consider guilt as only an emotional state (e.g., the state of guilt high or low at the moment), this finding is consistent with previous studies which also suggest that the narcissism is immune to feelings of guilt (Giammarco & Vernon, 2015; Wright et al., 1989). The subscales of guilt proneness are related to tendencies usually arising in response to personal transgressions in private (e.g., “I did something bad.” Response: repair the negative behaviour) (Cohen et al., 2011). Accordingly, guilt as a moral and negative emotion is associated with a private sense of having acted in a manner that violates one’s conscience. The negative relationship between narcissism and guilt proneness may be related to the lack of empathy and excessive sense of entitlement which often characterizes narcissism (American Psychiatric Association, 2013). According to other studies, guilt proneness is positively correlated to empathy (Torstveit et al., 2016; Xu et al., 2012). Individuals who are high in narcissism usually report negative associations to empathy, and it may reflect their inability to take other individual's emotional state, which also often may lead to antisocial behaviour (Delič, Novak, Kovačič, & Avsec, 2011; Eisenberg, 2007; Karremans, Van Lange, & Holland, 2005; Vonk, Zeigler-Hill, Mayhew, & Mercer, 2013). The sense of entitlement is considered as a salient trait among narcissistic individuals, through an unreasonable expectation of favourable treatment. They expect to be catered to, and may become aggressive if this does not happen (American Psychiatric Association, 2013). Accordingly, it might be a reason why they are not able to take into account the needs and desires of others.

In terms of personality, individuals with high guilt NBE (negative behaviour evaluation) scores are more prone to be more empathic, humble, conscientious, agreeable, and altruistic than those with low guilt NBE scores. In addition, individuals who are high in guilt NBE are more likely to show a desire to repair their behaviour and to prevent future negative behaviour (Cohen et al., 2011).

### Shame Proneness in Narcissistic Individuals

The current study indicates different responses among both dimensions of narcissism regarding shame proneness’ subtypes. There was a negative correlation between the grandiose narcissism and shame NSE (negative self-evaluation), while there were no significant findings between vulnerable narcissism and shame NSE. However, this study suggests that there is a positive correlation between vulnerable narcissism and shame withdraw, but there was no significant relation between grandiose narcissism and shame withdraw. Previous studies confirm a positive relation between the vulnerable narcissism and shame (e.g., shame as an emotional state) (Freis et al., 2015). Shame proneness (negative self-evaluations and withdraw), arising in response to personal transgressions in public (e.g., “I’m a bad person.” Response: Withdraw or hiding behaviour from public). Furthermore, shame is a negative emotion, often elicits when one’s transgressions are exposed publicly (Cohen et al., 2011). The fact that the grandiose narcissism showed a negative correlation with shame NSE, may be explained by grandiose narcissism’s high levels of self-esteem, unlike the vulnerable narcissism. Self-esteem is usually defined as a psychological term that goes back to the subjective evaluations that a person makes on one’s self – worth (Hewitt, 2009). Some studies show that both dimensions of narcissism can vary in their self-esteem, where vulnerable narcissism is usually associated with low levels of self-esteem in contrast to the grandiose dimension of narcissism (Wright et al., 1989). On the basis of previous studies, shame NSE is strongly related to low self-esteem, more than guilt NBE (negative behaviour evaluation) (Cohen et al., 2011; Tangney & Dearing, 2002). The current study, on the other hand shows a positive correlation between the vulnerable narcissism and shame withdraw. It can be explained by vulnerable narcissisms sensitivity to criticism, to others' evaluations, and low self – esteem (Cain et al., 2008). In addition, there may be a reason why the vulnerable narcissism tends to hide their negative behaviour from the public (e.g., fear to criticism). As with guilt NBE, individuals with lower shame NSE scores are more likely to make unethical decisions, commit delinquent behaviours, and lie for monetary gain (Cohen et al., 2011).

### Private and Public Distinctions of Moral Emotions and Influences on Ethical – or Unethical Behaviour

An important perspective to understand are the distinctions between private and public moral emotion tendencies. Guilt NBE (negative behaviour evaluation) is more linked to private evaluations of behaviour that violates one’s moral standards (Combs, Campbell, Jackson, & Smith, 2010; Smith, Webster, Parrott, & Eyre, 2002). Both of vulnerable and grandiose narcissism show a negative association with guilt NBE. Shame NSE (negative self-evaluation) in the other side, is more connected to evaluations of one’s publicly actions and behaviours that violates general ethical standards (Combs et al., 2010; Smith et al., 2002). Grandiose narcissism is negatively associated with shame NSE, while there was no significant association between the vulnerable narcissism and shame NSE. Thus, both vulnerable and grandiose narcissistic individuals tend to have dysfunctional moral standards which are inconsistent with acceptable moral and ethical ideals (e.g., moral: more related to personal perception of right and wrong; ethic: more related to common and social standards of right and wrong). This inability increases the tendency and frequency to commit delinquent offenses, which may lead to destructive consequences for the community (Haidt & Kesebir, 2010).

The present findings indicate that guilt proneness (measured by negative behaviour evaluation and repair items) and shame proneness (negative self-evaluation and withdraw items) explained together 20% of the variance in the vulnerable narcissism, whereas the same predictors only explained 11% of the difference in the grandiose narcissism. Tangney, Stuewig, Mashek, and Hastings (2011) suggest that guilt - and shame proneness have different influences on the self and behaviour. Individuals who score positive and high in guilt proneness, report lower levels of antisocial personality disorder and criminogenic cognitions. In contrast, individuals who report high and positive levels of shame proneness are positively connected to antisocial personality disorder and criminogenic cognitions (Tangney et al., 2011). Cohen et al. (2011) discuss the importance of differentiation of moral emotions and their different effect on behaviour. Shame proneness is positively associated with psychological dysfunctioning (e.g., neuroticism, personal distress), while guilt proneness is negatively correlated with unethical decision making (Cohen et al., 2011). Since there are different influences that both guilt and shame proneness have on ethical behaviour - in future research, it might be furthermore useful to observe the variance of narcissism by guilt and shame proneness separately (e.g., grandiose narcissism and guilt proneness; grandiose narcissism and shame proneness etc.), and not together.

Results of a mediation analysis suggest that the grandiose dimension of narcissism would predict the ethical behaviour (negative relation), by guilt repair with 4%. On the other hand, grandiose narcissism and guilt NBE (negative behaviour evaluation) together would predict ethical behaviour (negative correlation) with 42%. The same results apply also in vulnerable grandiose and ethical behaviour, by guilt repair (see the Figure 3 and 4 in the result section). When it comes to unethical behaviour - the findings of this study showed different results between the grandiose - and vulnerable narcissism. Vulnerable narcissism would predict unethical behaviour, by shame withdraw with 13% - and with a mediator, shame NSE (negative self-evaluation), together would predict unethical behaviour with also 13%. However, the grandiose narcissism and unethical behaviour by shame withdraw had no significant relation together. The last result may reflect the differences in personality between grandiose and vulnerable narcissism, and their different responses to social circumstances.

The distinction between the grandiose and vulnerable narcissism in regard to their proneness to experience moral emotions, is of a great importance of both theoretical and practical approach. More research is needed to illuminate differences between these two dimensions of narcissism as two separately personality patterns; particularly in which traits they differ from each other; and how they tend to act in social contexts. The present study confirms the notion that the grandiose and vulnerable narcissism differ in their personality types. Although both tend to have inability to experience guilt proneness and thus may not be able to repair unethical behaviour, it seems that vulnerable narcissism, in addition, tend to withdraw the unethical behaviour from public. This kind of knowledge may be important in job recruitment, e.g., employment of trustworthy people, and also in identification of unethical behaviour and tendencies in individuals with narcissistic traits.

### Limitations and Suggestions for Future Research

Because of the study’s electronic participation in an online survey, the selection attracted and consisted of many different age groups. The present study included low age of participants, and age range started from age 15. This particular issue may raise opportunities for new discussions; whether the sample is representatively enough for the normal population, since the phenomenon narcissism begins by early adulthood (e.g., according to American Psychiatric Association, 2013). To avoid such mistakes in future research, it will be more appropriate to inform participants that the desired age for participation is from age of 18.

Some of guilt and shame proneness subscales of our sample had poor reliability. Especially shame withdraw (.506) and guilt repair (.660), due to the low number of items (four items) but these were similar to the original scale from Cohen et al. study (2011), and also other studies which also reported low Cronbach’s alpha (e.g., Arli, Leo, & Tjiptono, 2016). This lower reliability compared to the original scale could be caused by the heterogeneous (18 different countries) sample compared to the original scale (one country).

Another limitation of the study was the educational background of the participants. The majority of the respondents seemed to be highly educated. This can also limit the variation in the sample, and therefore may not be representative for other social groups. In addition, the gender of this study was imbalanced, with 74.5% female. This restriction may be regarded as a less significance for the result of the study, because the phenomenon narcissism is usually gender independent, as other studies confirm (e.g., Reinhard, Konrath, Lopez, & Cameron, 2012). The sample size of this study consists of less than 250 participants (*N* = 216). According to Schönbrodt and Perugini (2013), it can be problematic for stable estimates. Usually correlations stabilize when *N* approaches 250 (Reinhard et al., 2012). In addition, since this study presents two sub-groups of narcissism, there is more need for a larger sample size to verify the validity of these findings and to avoid method-bias. Future research in this area must therefore ensure that population size achieve at least 250 participants to secure the stability in correlations. Some values of mediation analysis achieved significant values, *p* <.05. According to Goodman (2001), *p* <.05 have a high probability of acceptance of null hypothesis up to 30%. Therefore, this level of *p* values must be reported as one of the limitations of this study.

Hepper, Hart, and Sedikides (2014) found that narcissistic individuals are able to take another's emotional state at perspective-taking, by using a concrete example (e.g., items that are not instructional, but more illustrative and based on a specific situation). Since there is a positive relation between empathy by perspective-taking and moral emotions (e.g., guilt) - future research may aim to investigate guilt, shame proneness and narcissism in more specific cases (e.g. job context) with perhaps same reformulations of the GASP items. With a concrete example, it may be possible to observe whether narcissistic individuals still make unethical decisions. This kind of research can be interesting in job recruitment and may avoid unethical behaviour in job contexts (e.g., cheating).

### Conclusion

This study is among the first to examine the roles of shame - and guilt proneness on the tendency to act ethically in individuals with narcissistic traits. Findings of this research indicate that there were a negative association between guilt proneness and vulnerable narcissism, and also a negative relation between grandiose narcissism and guilt proneness. In other words, both of vulnerable and grandiose narcissism are unable to take into account their unethical behaviour as negative tendencies for the community, and also unwillingness to change their unethical behaviour. In addition, we found no significant association between vulnerable narcissism and the subscale shame negative self-evaluation. This finding was inconsistent with our expectations. Finally, the present research suggests a positive association between vulnerable narcissism and shame withdraw. This result suggests that individuals high in vulnerable narcissism may be more prone to conceal behaviour which transgress social norms and moral.

Future research in the area should include potential influences of moral emotions on behaviour, more specifically how shame and guilt proneness can influence the tendency to act unethically in individuals with narcissistic traits.
